# Serum Levels of Soluble ST2 and IL-10 Are Associated with Disease Severity in Patients with IgA Nephropathy

**DOI:** 10.1155/2016/6540937

**Published:** 2016-11-09

**Authors:** Zhihui Zhang, Haifeng Wang, Li Zhang, Rebecca Crew, Nan Zhang, Xiaolei Liu, Yanfang Jiang

**Affiliations:** ^1^Genetic Diagnosis Center, The First Hospital of Jilin University, Changchun 130021, China; ^2^Key Laboratory of Zoonoses Research, Ministry of Education, The First Hospital of Jilin University, Changchun 130021, China; ^3^Department of Nephrology, The First Hospital of Jilin University, Changchun 130021, China; ^4^University of Oklahoma Health Sciences Center, Oklahoma City, OK 73104, USA; ^5^Jiangsu Co-Innovation Center for Prevention and Control of Important Animal Infectious Diseases and Zoonoses, Yangzhou 225009, China

## Abstract

*Background*. The IL-33/ST2 axis is involved in humoral immune responses.* Method*. The concentrations of sera IL-33 and sST2 in 74 patients and 34 healthy controls (HC) were measured by ELISA. Clinical and laboratory data were examined. The potential association between sera IL-33 and sST2 and the clinical parameters in IgAN patients were analyzed.* Results*. No difference was discovered in IL-33 concentrations between IgAN patients and HCs; however, the sST2 were significantly higher in each stage of IgAN progression than in the HC. The concentration of sST2 was positively correlated with IL-33 levels in IgAN patients. Higher levels of sera IL-2, IL-4, IL-10, IL-17A, and IFN-*γ* were detected in patients compared to the HC. The concentration of serum sST2 was positively correlated with the levels of IL-10 in IgAN patients. Furthermore, serum sST2 was negatively correlated with the values of eGFR and serum calcium. Serum sST2 was positively correlated with 24-hour urinary protein, serum phosphorus, and serum IgA; however, serum IL-33 was not associated with these. Following treatment, serum sST2 was significantly decreased, while sera IL-4 and IL-10 were significantly increased.* Conclusions*. Increased sST2 and IL-10 but not IL-33 may be involved in the pathogenic process of IgAN.

## 1. Introduction

IgA nephropathy (IgAN) is the most common form of glomerulonephritis (GN), constituting 30–45% of all primary glomerular diseases in Asians and about 30–40% in Caucasians, whereas the prevalence is lower in Blacks [1, 2]. IgAN is characterized by mesangial deposition of predominantly IgA alone or with IgG, IgM, or complement C3 in the glomeruli of the kidney [2]. Up to 20–40% of patients have a slow progressive disease, resulting in end stage renal disease (ESRD) 10 years after the original diagnostic renal biopsy [3, 4]. At present, the nosogenesis of IgAN is not fully comprehended. Prevenient researches have shown that some genetic factors are connected with the evolution of IgAN [5]. Recent studies indicate that IgAN is an immune complex-mediated nephritogenic immune response, yet the pathogenesis of IgAN remains unclear. It is imperative to develop biomarkers for early diagnosis and discrimination of IgAN severity [6, 7].

Interleukin-33 (IL-33), a cytokine of the IL-1 family, is expressed in epithelial barrier tissues and lymphoid organs and plays important roles in type-2 innate immunity, human asthma, and parasitic infection. IL-33 functions as an “alarmin,” activating various immune cells through its receptor ST2, which brings about a variety of molecules and cytokines. ST2 is expressed on various cells including hematopoietic cells such as mast cells, basophils, eosinophils, Th2 cells, macrophages, dendritic cells, NK cells, and type 2 innate lymphoid cells (ILC2), but not Th1 cells [8]. ST2, called IL-1RL1 and T1, is known to exist in a transmembrane form (ST2L) and in a secreted soluble form (sST2). sST2 serves as a decoy receptor by binding to free IL-33 and preventing its signaling through ST2L, while ST2 functions as a mediator of IL-33 bioactivities [9]. The IL-33/ST2 axis appears to play a pivotal role in some chronic immune inflammatory diseases including asthma [10], rheumatoid arthritis [11], and anaphylactic shock [12]. sST2 is an important marker and its level is related to the severity of these diseases [11, 13]. However, there is currently no information on whether the IL-33/ST2 axis participates in the pathogenesis of IgAN.

Recent studies have indicated that the urinary excretion levels of IL-2, IL-4, IL-6, IL-10, IL-17A, IFN-*γ*, and TNF-*α* were significantly elevated in patients with IgAN compared to healthy controls [14]. Another study found that the mRNA levels for IL-4, IL-2, and IFN-*γ* of CD4^+^ cells were increased in the patients with IgAN compared to the HC [15]. However, little is known about the change of these serum cytokines in IgAN patients.

In our study, we detected the serum levels of IL-33 and sST2 in patients with IgAN and healthy controls. Furthermore, we made a thorough inquiry into the relationship between serum sST2 and the severity of the disease. Our research may provide new insights into the function of the IL-33/ST2 axis in the pathogenesis of IgAN.

## 2. Materials and Methods

### 2.1. Patients

A total of 74 patients with IgAN were recruited from the in-patient service of the First Hospital of Jilin University (Changchun, China) from July 2012 to August 2015. Written informed consent was gained from individual participants. Individual patients with IgAN were diagnosed on the basis of the World Health Organization (WHO) histological classification standards [16]. The WHO classification uses the following categories. Stage I: the masses of glomeruli are normal by light microscopy. The lesions are defined as minimal changes. Stage II: >50% of glomeruli are normal and a small proportion shows localized mesangial proliferation, whereas sclerosis, adhesions, and small crescents are rare. Tubular and interstitial lesions are absent. Stage III: diffuse mesangial proliferation and thickening with focal and segmental variations are visible. Adhesions and small crescents are occasionally seen. Focal interstitial edema and infiltrates are occasionally present. Tubular atrophy is rare. Stage IV: almost all glomeruli show marked diffuse mesangial proliferation and sclerosis with altering degrees of hypercellularity and random lesion distribution. Variable numbers of obsolescent glomeruli are frequently seen. Up to 50% of glomeruli contain adhesions and crescents. Tubular atrophy and interstitial inflammation are obvious. Stage V: partial and/or global sclerosis, hyalinosis, and capsular adhesions are seen; glomeruli containing adhesions and crescents >50%. Lesions are characterized by diffuse sclerosing glomerulonephritis. Furthermore, tubular and interstitial variations are more severe than those in grade IV.

The experimental protocol was designed according to the guidelines of the Declaration of Helsinki and was ratified by the Human Ethics Committee of First Hospital of Jilin University. Some of the IgAN patients proved to have nephritic syndrome according to the diagnostic criteria, including massive proteinuria (>3.5 g/day), hypoalbuminemia (albumin < 30 g/L), hyperlipidemia, and edema. Hormone drugs or other immunosuppressive agents were not used to treat any patient prior to our research. To select appropriate patients, all were screened for ANA, anti-Sm antibody, anti-SSA, anti-SSB, anti-neutrophil cytoplasmic antibody (ANCA), rheumatoid factor, viral serology, and blood glucose, in addition to an ophthalmological review or echocardiogram. Exclusion criteria include (1) rapidly progressive IgAN (with fast renal function deterioration or historically necrotic capillaries) and secondary IgAN, such as lupus nephritis, Henoch-Schonlein purpura, and diabetes mellitus, and (2) presence of malignancy, pregnancy, neoplasia, active peptic ulcer disease, and chronic infection. Another 34 age- and gender-matched healthy controls were enrolled at the Physical Examination Center of the same hospital during the same period. Demographic and clinical characteristics were recorded and analyzed (Table 1).

### 2.2. Treatment and Follow Up

Individual patients were treated with prednisolone (PDN, Tianyao Pharmaceuticals, Tianjin, China) at 1 mg/kg/day dosage for the first two months, which was then gradually tapered to a maintenance dose of 10 mg/day dosage over the next six-month duration. They were also treated with immunosuppressant at the same time. The patients were followed up for 8 to 12 weeks. There were altogether 9 patients with complete records and the other 65 patients failed to follow up. Among the 9 patients, 4 were in Stage I+II, 3 were in Stage III, and 2 were in Stage IV+V. After 8–12 weeks of treatment, the serum samples were gathered at the time of kidney biopsy.

### 2.3. Measurement of Sera IL-33 and sST2 by Enzyme-Linked Immunosorbent Assay (ELISA)

The concentrations of sera IL-33 and sST2 in IgAN patients and healthy controls were measured by ELISA using human IL-33 (affymetrix ebioscience, California, USA) and sST2 (ImmunoWay, California, USA) ELISA kit, according to the manufacturer's instruction. The concentrations of sera IL-33 and sST2 in individual samples were calculated according to the standard curve established using the recombinant IL-33 and sST2 provided. The detection limit for human IL-33 and sST2 was 0.2 pg/mL and 0.25 pg/mL, respectively.

### 2.4. Cytometric Bead Array (CBA) Analysis of Serum Cytokines

The concentrations of total sera IFN-*γ*, TNF-*α*, IL-2, IL-4, IL-6, IL-10, and IL-17A were determined by CBA [17], according to the manufacturer's protocol (BD Biosciences) with minor modifications. The individual sera (25 *μ*L/each) were detected in duplicate on a FACSCalibur cytometer (BD Biosciences) [18], and the concentrations of serum cytokines were quantified using the CellQuest Pro and CBA software (Becton Dickinson) on a BD FACS Aria II.

### 2.5. Statistical Analysis

All data are expressed as median and range. The difference between two groups was analyzed by the Mann-Whitney* U* nonparametric test. The relationship between variables was evaluated using the Spearman's rank correlation test. All statistical analyses were performed by the SPSS 19.0 software. A two-sided *p* value of <0.05 was considered statistically significant.

## 3. Results

### 3.1. Characteristics of IgAN Patients

To examine the potential role of the concentrations of sera IL-33 and sST2 in the development of IgAN, a total of 74 Chinese patients with newly diagnosed IgAN and 34 age- and gender-matched healthy controls were recruited. No significant differences were detected in the distribution of age and gender between the patients and HC. Furthermore, we found no significant difference in the concentrations of serum uric acid, triglycerides, cholesterol, and urea nitrogen and the numbers of lymphocytes between these two groups of subjects (Table 1). As expected, there was a marked increase in the concentrations of serum IgA, phosphorus, microscopic hematuria, and 24 h urinary proteins in the patients compared with the HC. However, the values of eGFR, serum calcium, and serum albumin in the patients were notably less than in the HC. These data suggest that those patients had abnormal IgA responses and kidney function lesions.

### 3.2. Serum Levels of IL-33 and sST2

**Figure 1:**
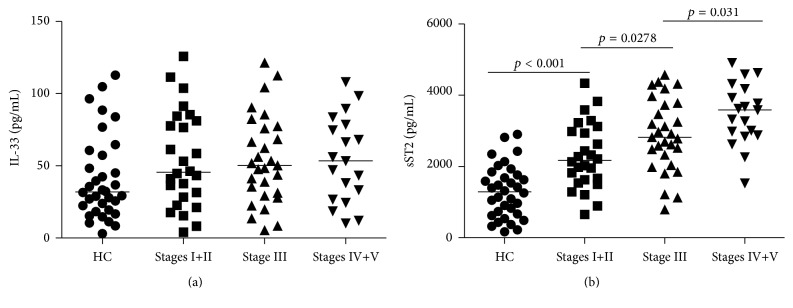
Analysis of sera IL-33 and sST2 in IgAN patients and HC. The levels of sera sST2 and IL-33 in IgAN patients and HCs were tested by ELISA. Data are expressed as the mean values of individual participants from two separate experiments; horizontal lines represent the median values of different groups. (a) Analysis of serum IL-33 levels revealed no obvious differences among different stage IgAN patients and healthy controls (HC) (all *p* > 0.05). No significant difference in the levels of serum IL-33 was found among each stage of IgAN patients. (b) Serum sST2 levels were significantly higher in each stage of IgAN patients than in HC and furthermore tended to increase in parallel with the severity of the histopathological classification of IgAN (*r* = 0.443, *p* < 0.001).

Serum levels of IL-33 and sST2 were tested in 74 patients and 34 age- and sex-matched healthy individuals. Analysis of serum cytokines indicated no significant difference in the levels of serum IL-33 between patients with IgAN at each stage compared to the HC (all *p* > 0.05, Figure 1(a)). There was no significant difference in the concentrations of serum IL-33 between the different stages of IgAN in patients (data not shown), while the levels of serum sST2 were higher in each stage of IgAN patients than in HC (all *p* < 0.05, Figure 1(b)). Furthermore, the serum sST2 tended to increase in parallel with the severity of the histopathological classification of IgAN (*r* = 0.443, *p* < 0.001, Figure 1(b)).

### 3.3. Correlation Analysis of Serum sST2 with the Levels of IL-33 in IgAN Patients

**Figure 2:**
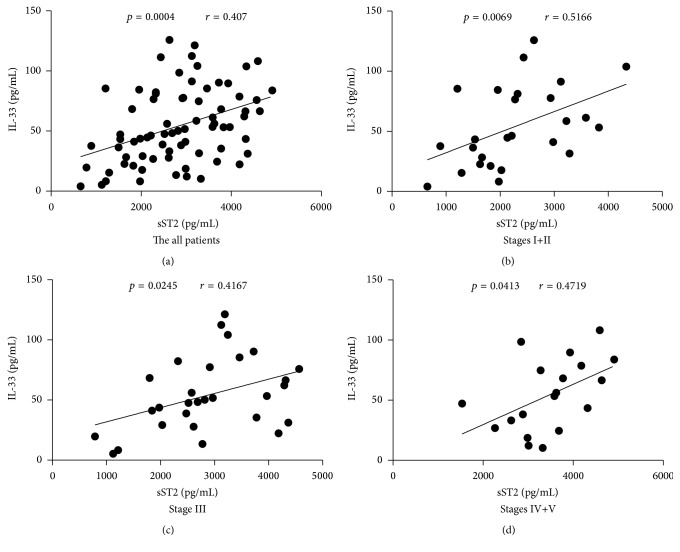
Correlation analysis of serum sST2 with IL-33 levels in IgAN patients. The potential relationships between sera sST2 and IL-33 levels in IgAN patients were analyzed by Spearman correlation tests. Data shown are the mean concentration of individual subjects from two separate experiments. ((a)–(d)) Serum sST2 was positively correlated with IL-33 levels in all patients and also in Stage I+II, Stage III, and Stage IV+V of IgAN patients.

In order to understand the relationship between serum sST2 and IL-33, we explored the potential association of serum sST2 with levels of IL-33 in patients. We found that serum sST2 was correlated positively with the levels of IL-33 in all patients (*r* = 0.407, *p* = 0.0004, Figure 2(a)), and also in Stage I+II (*r* = 0.5166, *p* = 0.0069, Figure 2(b)), Stage III (*r* = 0.4167, *p* = 0.0245, Figure 2(c)), and Stage IV+V (*r* = 0.4719, *p* = 0.0413, Figure 2(d)) of IgAN patients.

### 3.4. The Correlation between Serum sST2 and the Values of Clinical Parameters in IgAN Patients

**Figure 3:**
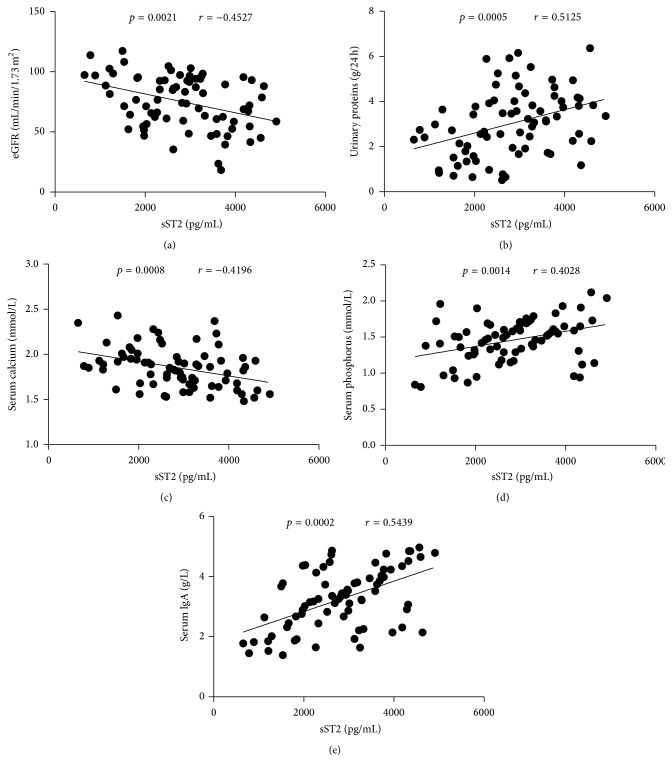
Correlation analysis of serum sST2 with the values of clinical parameters in IgAN patients. The potential correlations between serum sST2 and the values of clinical parameters were analyzed by Spearman correlation tests. Data shown are the mean concentration of individual subjects from two separate experiments. (a) Serum sST2 levels were correlated negatively with the values of eGFR. (b) Serum sST2 levels were correlated positively with the concentrations of 24-hour urinary proteins. (c) Serum sST2 levels were correlated negatively with serum calcium. ((d)-(e)) Serum sST2 levels were correlated positively with serum phosphorus and serum IgA, respectively.

To understand the importance of sST2 in the pathogenesis of IgAN, we analyzed the potential relationship of serum sST2 with the values of clinical measures detected in these patients. We found that the serum sST2 levels were correlated negatively with the values of eGFR (*r* = −0.4527, *p* = 0.0021, Figure 3(a)) and serum calcium (*r* = −0.4196, *p* = 0.0008, Figure 3(c)); however, they correlated positively with the concentrations of 24-hour urinary proteins (*r* = 0.5125, *p* = 0.0005, Figure 3(b)), serum phosphorus (*r* = 0.4028, *p* = 0.0014, Figure 3(d)), and serum IgA (*r* = 0.5439, *p* = 0.0002, Figure 3(e)) in these patients. No obvious correlation was found between clinical index and IL-2, IL-4, IL-10, IL-17A, IFN-*γ*, and IL-33 levels (data not shown). Together, this data suggests that sST2 may participate in the mechanism of IgAN and play an important role in the disorder of bone pathogenesis in IgAN patients.

### 3.5. Elevated Serum Levels of IL-2, IL-4, IL-10, IL-17A, and IFN-*γ* in IgAN Patients before Treatment

**Figure 4:**
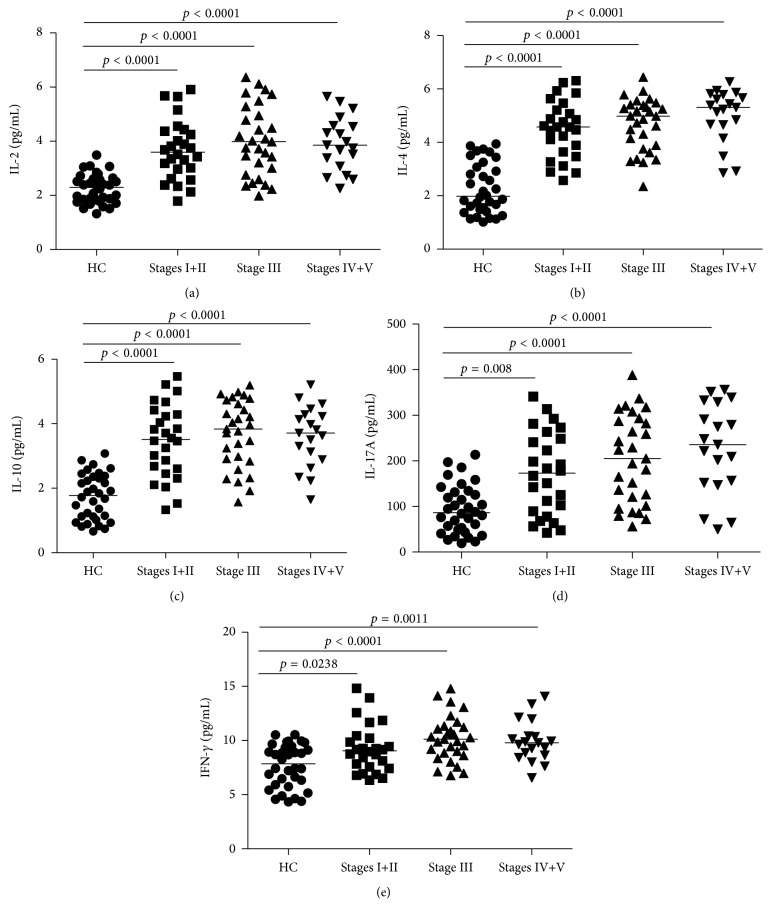
Increased serum levels of IL-2, IL-4, IL-10, IL-17A, and IFN-*γ* in IgAN patients before treatment. CBA was used to detect the levels of sera IL-2, IL-4, IL-17A, IL-10, and IFN-*γ* ((a)–(e), resp.) in IgAN patients and HCs. Data are expressed as mean values of individual samples from two divided experiments. Horizontal lines represent median values of different groups.

To explore the potential function of IL-2, IL-4, IL-10, IL-17A, and IFN-*γ* in IgAN, we measured the serum levels by cytometric bead array (CBA). We found that the concentrations of sera IL-2, IL-4, IL-10, IL-17A, and IFN-*γ* were significantly increased in IgAN patients compared to the HCs (*p* < 0.05, Figures 4(a)–4(e)). No apparent differences in the concentrations of those cytokines were discovered among the different stages of IgAN patients (data not shown). Our data indicates that those cytokines may participate in the pathogenesis of IgAN.

### 3.6. Correlation Analysis of Serum sST2 with the Levels of IL-2, IL-4, IL-10, IL-17A, and IFN-*γ* in IgAN Patients

**Figure 5:**
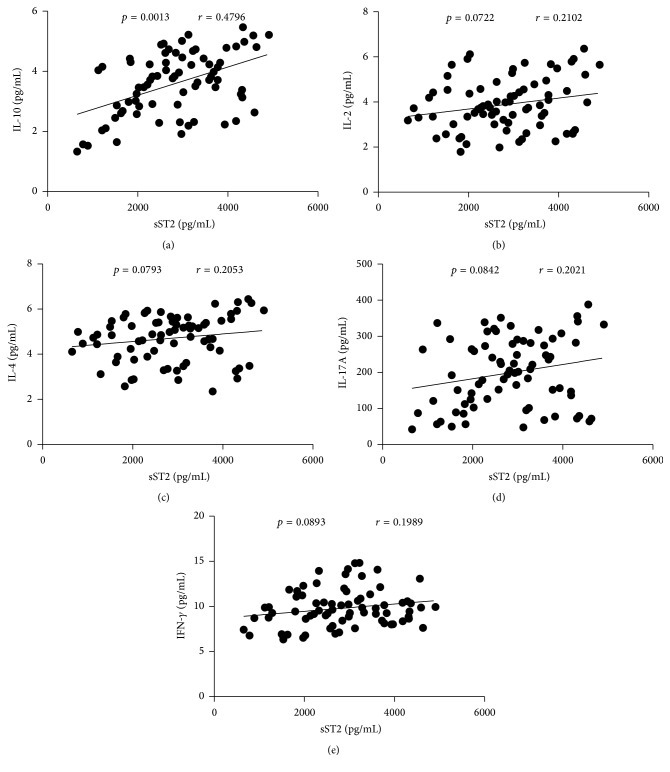
Correlation analysis of serum sST2 with the levels of IL-2, IL-4, IL-10, IL-17A, and IFN-*γ* in IgAN patients. The potential correlations between serum sST2 and the levels of IL-2, IL-4, IL-10, IL-17A, and IFN-*γ* were analyzed by Spearman correlation tests. Data shown are the mean concentration of individual subjects from two separate experiments. (a) Serum sST2 levels were correlated positively with IL-10. ((b)–(e)) No correlation was found between serum sST2 and the levels of IL-2, IL-4, IL-17A, and IFN-*γ* in IgAN patients, respectively.

To comprehend the importance of sST2 in the pathogenesis of IgAN, we explored the potential association of the serum sST2 with the levels of IL-2, IL-4, IL-10, IL-17A, and IFN-*γ* in patients. We found that serum sST2 correlated positively with the level of IL-10 (*r* = 0.4796, *p* = 0.0013, Figure 5(a)), whereas there was no significant correlation between serum sST2 and the levels of IL-2, IL-4, IL-17A, and IFN-*γ* in IgAN patients (Figures 5(b)–5(e)).

### 3.7. Correlation Analysis of Serum sST2 with the Levels of IL-10 in the Different Stages of IgAN Patients

**Figure 6:**
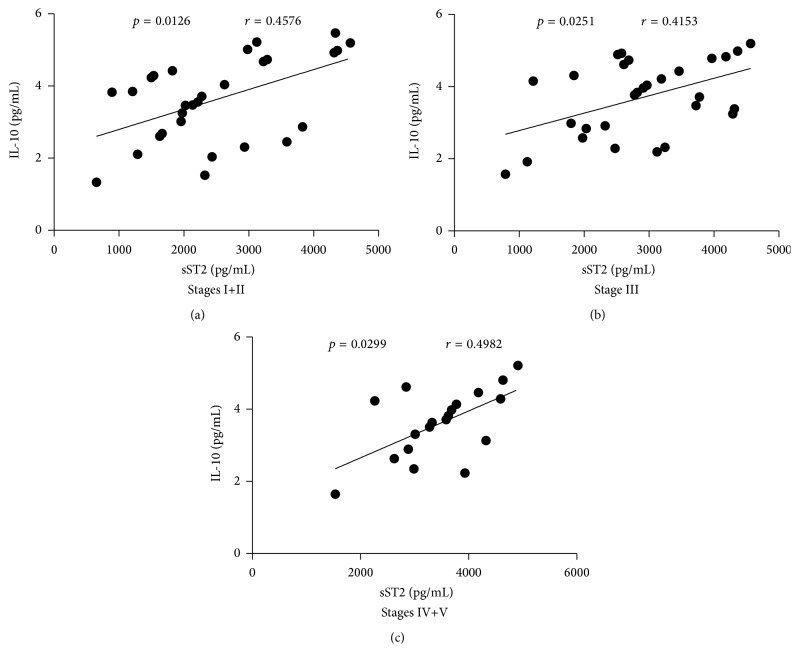
Correlation analysis of serum sST2 with the levels of IL-10 in different stages of IgAN patients. The potential relationships between serum sST2 and the levels of IL-10 in the different stages of IgAN patients were analyzed by Spearman correlation tests. Data shown are the mean concentration of individual subjects from two separate experiments. ((a)–(c)) Serum sST2 was positively correlated with the levels of IL-10 in Stage I+II, Stage III, and Stage IV+V of IgAN patients, respectively.

Since Figure 5(a) showed that serum sST2 was positively correlated with IL-10 levels (*r* = 0.4796, *p* = 0.0013), we further analyzed the correlations between serum sST2 and the levels of IL-10 in different stages of IgAN patients. We discovered that serum sST2 was positively correlated with the levels of IL-10 in Stage I+II (*r* = 0.4576, *p* = 0.0126, Figure 6(a)), Stage III (*r* = 0.4153, *p* = 0.0251, Figure 6(b)), and Stage IV+V (*r* = 0.4982, *p* = 0.0299, Figure 6(c)) of IgAN patients.

### 3.8. Clinical Character and the Levels of Sera Cytokines in IgAN Patients following Treatment

**Figure 7:**
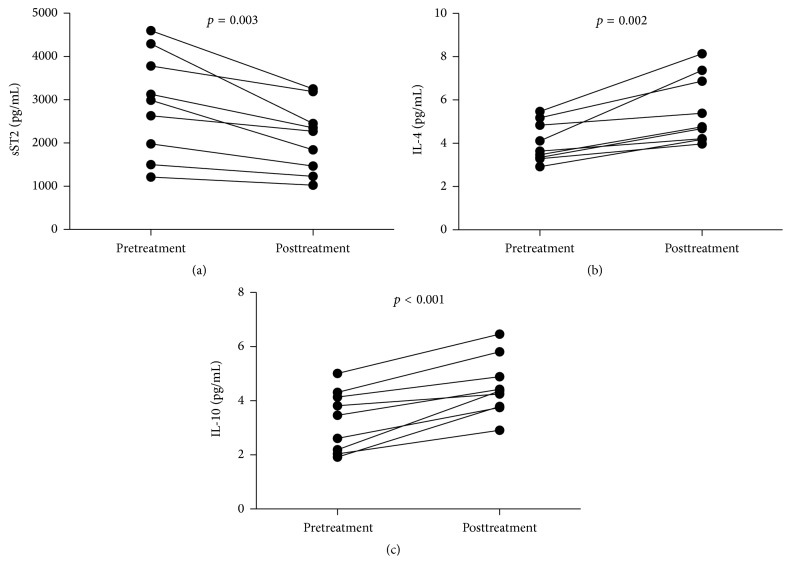
Levels of serum cytokines in IgAN patients following treatment. The concentrations of serum cytokines were compared in IgAN patients before and after treatment. The data are expressed as the mean % or concentration of individual subjects from two divided experiments. ((a)–(c)) The levels of serum of sST2, IL-4, and IL-10 in individual patients pre- and posttreatment, respectively.

In order to better understand the function of the IL-33/ST2 axis in the development of IgAN, we analyzed the values of clinical parameters and levels of sera cytokines in 9 patients, who were followed up for 8–12 weeks. There were altogether 9 patients with integrated records, while the other 65 patients failed to follow up. We found that the values of 24-hour urinary protein, microscopic hematuria, serum IgA, and serum phosphorus were significantly descending but the values of eGFR and serum calcium were significantly elevating in the patients (Table 2). Furthermore, the level of serum sST2 was significantly decreased compared to the pretreatment levels (*p* = 0.003, Figure 7(a)). However, the levels of sera IL-4 (*p* = 0.002, Figure 7(b)) and IL-10 (*p* < 0.001, Figure 7(c)) were increased compared to pretreatment levels. There was no significant difference in the levels of other serum cytokines detected before and after treatment (data not shown). Collectively, treatment significantly improved proteinuria and lessened the level of serum sST2 but elevated inhibitory IL-4 and IL-10 responses in patients with IgAN.

## 4. Discussions

IL-33 is a polymorphous cytokine that participates in diversified disease conditions [13, 19, 20]. IL-33 can activate the MAP kinase and NF-*κ*B signal pathways and facilitate Th2 response and cytokine generation by its receptor complex composed of ST2 and IL-1RaP [10]. Endogenous IL-33 is conducive to airway inflammation, and intranasal control of IL-33 triggers an instant allergic response inside the airway [21]. One study discovered that serum IL-33 levels were increased in SLE and RA patients and were correlated with the levels of serum ESR and CRP, two inflammation markers, thereby suggesting that IL-33 may take part in the acute-phase response of SLE [22]. In addition, IL-33, through strengthening neutrophil permeation at the site of inflammation, can protect against septic shock [23]. The role of IL-33/ST2 axis in patients with IgAN remains unknown; therefore, it is of clinical importance to investigate the characteristics of IL-33 and ST2 in IgAN patients. The present study is the first to measure serum levels of IL-33 and sST2 and reveal the correlation between the IL-33/ST2 axis and disease severity in IgAN patients. However, no significant elevation of serum IL-33 and no significant affiliation between serum IL-33 level and disease severity were observed in IgAN patients. Previously, sST2 has been shown to capture IL-33 and acts as an antagonistic decoy receptor for IL-33 [24]. In this study, the serum IL-33 was correlated positively with the levels of sST2 in IgAN patients. These data suggest that IL-33 may not participate in the nosogenesis of IgAN or may be restrained by sST2 by negative regulatory mechanisms.

ST2 is selectively expressed on a subset of Th2 cells, but not Th1 cells, and participates in Th2 functions [8]. What is more, recent studies verify that sST2 can serve as a negative modulator and antagonistic decoy receptor for IL-33 [24, 25]. It has been shown that serum sST2 levels are higher in chronic immune inflammatory diseases than in healthy controls [26–28]. IgAN is a type of emblematic chronic immune inflammatory diseases. Our study shows higher sST2 levels in IgAN patients than in HC and reveals that serum sST2 levels are positively correlated with the severity of IgAN. Following treatment, the level of serum sST2 was significantly reduced compared to pretreatment. Furthermore, we found that the level of serum sST2 had a positive correlation with the levels of 24-hour urinary proteins and serum IgA but was negatively correlated with eGFR values in these patients. These data suggest that the elevation of sST2 may be conducive to the pathogenesis of IgAN, and the level of serum sST2 may act as an underlying biomarker for assessing the disease development in IgAN.

Th cells are divided into Th1 and Th2 cells. It is generally known that Th1 cells secret IFN-*γ* and IL-2 and mainly promote cell-mediated immunity. Alternatively, Th2 cells produce IL-4 and IL-10, in addition to primarily boosting the production of antibody. Previous research has shown that IL-17A plays a pivotal role in the pathogenesis of host defenses, autoimmune responses, and chronic inflammatory diseases [29, 30]. Our studies show that the levels of sera IL-2, IL-4, IL-10, IL-17A, and IFN-*γ* in IgAN patients were significantly elevated compared to the HC. Furthermore, treatment noticeably increased the levels of sera IL-4 and IL-10 but did not affect the levels of sera IL-2, IL-17A, and IFN-*γ*. Our data suggest that proinflammatory Th1 and Th17 responses may be involved in the mechanism of IgAN and excite anti-inflammatory Th2 and Tregs that then feedback to downregulate proinflammatory responses during the pathogenesis of IgAN.

It is known that sST2 can bind to IL-33 as a decoy molecule to promote anti-inflammatory effects [24]. In addition, IL-10 is an anti-inflammatory factor. Previous research has shown that the protective effect of sST2 does not persist in IL-10 knockout mice, suggesting that IL-10 is necessary for the beneficial effect of sST2 [31]. Significantly, we demonstrated a positive correlation between serum sST2 concentration and IL-10 levels in IgAN patients. These data suggest that sST2 must likely work in cooperation with IL-10 to exert its function in IgAN patients.

Our studies showed that the levels of serum calcium were significantly decreased and the levels of serum phosphorus were significantly increased in IgAN patients before treatment, compared to HC. Furthermore, serum sST2 was correlated positively with serum phosphorus but negatively with serum calcium. After treatment, the levels of serum calcium were significantly elevated and the levels of serum phosphorus were significantly decreased. FGF-23, an osteocyte-derived hormone, can effectively stimulate renal phosphorus excretion and concomitantly inhibits PTH [32]. One study found that FGF-23 can antagonize some effects of vitamin D* in vitro* [33]. SST2 is a target of PTH and it can aggrandize osteoblastic matrix mineral deposition and inhibit osteoclast formation [34]. According to some studies chronic kidney disease (CKD) patients with elevated PTH had a higher level of sST2 compared to HC [35]. IgAN is one of the most important CKDs. The concentrations of calcium and phosphorus maintain a relatively constant level in plasma of normal people, and when phosphorus is increased, serum calcium is reduced. PTH has the effect of elevating blood calcium and reducing blood phosphorus. These data suggest that sST2 may play an important role in the disorder of bone metabolism in IgAN patients.

In conclusion, our results indicate that the concentration of serum sST2 is significantly higher in IgAN patients, suggesting that sST2 may play a critical role in the pathogenesis of IgAN and may serve as a biomarker for evaluating disease severity. The limitations of our study include a small number of enrolled patients, single time-point measurement for some patients, little information from the affected kidney, and lack of research on sST2 after treatment. Although more detailed studies are necessary to define the regulatory mechanisms of sST2 in the pathogenic process in IgAN, our novel findings provide new insights into understanding the pathogenesis of IgAN and may expand therapeutic options for patients.

## Figures and Tables

**Table 1:** Demographic and clinical characteristics of participants.

	HC (*n* = 34)	IgAN patients (*n* = 74)		
		Stage I+II (*n* = 26)	Stage III (*n* = 29)	Stage IV+V (*n* = 19)
Age, year	43 (18–76)	37 (17–69)	46 (19–72)	42 (23–77)
Female/male	15/19	14/12	16/13	8/11
Lymphocytes, 10^9^/L	1.72 (0.84–2.87)	1.68 (0.87–3.22)	2.04 (0.82–4.13)	2.12 (0.89–3.92)
Serum albumin, g/L	43.2 (40.8–50.6)	35.3 (14.3–41.4)^*∗*^	33.2 (8.84–39.6)^*∗*^	30.24 (12.1–38.9)^*∗*^
Serum uric acid, *μ*mol/L	305 (245–416)	342 (231–604)	371 (252–611)	383 (247–584)
Triglycerides, mmol/L	1.27 (0.51–1.78)	2.32 (0.62–6.53)	2.56 (0.58–4.78)	2.23 (0.74–4.94)
Cholesterol, mmol/L	4.07 (2.86–5.47)	5.12 (3.12–8.78)	6.24 (3.23–9.14)	5.85 (2.86–10.05)
Urinary proteins, g/24 h	0.048 (0–0.12)	2.61 (0.65–4.13)^*∗*^	3.46 (0.52–6.36)^*∗*^	3.17 (0.71–5.89)^*∗*^
Urea nitrogen, mmol/L	5.14 (3.62–6.68)	5.95 (2.65–13.1)	6.46 (3.13–10.35)	6.87 (4.02–12.8)
eGFR, mL/min/1.73 m^2^	104.5 (88.5–112.4)	89.4 (41.6–117.3)^*∗*^	74.2 (45.1–113.8)^*∗*^	60.7 (18.3–102.9)^*∗*^
Microscopic hematuria, rbc/hpf	1.1 (0–2.4)	7.1 (0.2–23.4)^*∗*^	7.8 (0.8–27.6)^*∗*^	8.5 (1.2–19.4)^*∗*^
Serum calcium, mmol/L	2.38 (1.91–2.62)	1.91 (1.48–2.35)^*∗*^	1.82 (1.52–2.23)^*∗*^	1.86 (1.56–2.43)^*∗*^
Serum phosphorus, mmol/L	1.15 (0.74–1.49)	1.44 (0.84–1.91)^*∗*^	1.58 (0.81–2.12)^*∗*^	1.52 (0.93–2.04)^*∗*^
Serum IgA, g/L	1.87 (1.03–2.98)	3.16 (1.78–4.85)^*∗*^	3.34 (1.45–4.97)^*∗*^	3.52 (1.39–4.79)^*∗*^

Data shown are median and range, except specified. ^*∗*^ *p* < 0.05 versus the HC.

**Table 2:** Effect of treatment on the values of clinical measures in follow-up IgAN patients.

	Before treatment	After treatment
Age, year	46 (26–67)	46 (26–67)
Female/male	4/5	4/5
Lymphocytes, 10^9^/L	2.14 (1.24–3.65)	1.78 (1.11–3.21)
Serum albumin, g/L	29.7 (17.7–35.6)	37.8 (25.8–48.2)
Serum uric acid, *μ*mol/L	385 (263–583)	352 (231–516)
Triglycerides, mmol/L	2.82 (1.24–5.46)	2.55 (1.08–5.22)
Cholesterol, mmol/L	6.22 (3.28–9.26)	5.17 (2.11–7.13)
Urinary proteins, g/24 h	4.41 (0.94–5.26)	2.05 (0.38–3.32)^*∗*^
Urea nitrogen, mmol/L	5.71 (3.37–11.85)	4.62 (2.65–9.38)
eGFR, mL/min/1.73 m^2^	78.36 (48.74–102.85)	99.73 (60.26–118.38)^*∗*^
Microscopic hematuria, rbc/hpf	6.65 (1.35–21.12)	2.04 (0.21–10.23)^*∗*^
Serum calcium, mmol/L	1.81 (1.64–2.33)	2.37 (2.09–2.58)^*∗*^
Serum phosphorus, mmol/L	1.78 (1.03–2.08)	1.13 (0.81–1.45)^*∗*^
Serum IgA, g/L	3.27 (1.56–4.63)	1.82 (1.12–3.76)^*∗*^

Data are present as median (range). ^*∗*^ *p* < 0.05 versus values before treatment.
